# Trousseau's Syndrome With Non-bacterial Thrombotic Endocarditis Associated With Endometrioid Carcinoma in a Young Patient

**DOI:** 10.7759/cureus.104007

**Published:** 2026-02-21

**Authors:** Francisco David Roman-Delgado, Dhavyd Herrera-Ibañez, Corazon de Maria Sanchez-Martinez, Carlos Alejandro Tellez-Arellano, Carlos Eduardo Ortega-Jardinez, Antonio Quiñones-Soto, Fabian Enrique Rivera-Lopez, Luis Fernando Acevedo-Garcia

**Affiliations:** 1 Internal Medicine, Centro Médico Nacional Siglo XXI Mexican Social Security Institute (IMSS), Mexico City, MEX

**Keywords:** endometrioid cancer, hypercoagulability, ischemic stroke, non-bacterial thrombotic endocarditis, trousseau’s syndrome

## Abstract

Trousseau’s syndrome is a malignancy-associated hypercoagulable state characterized by recurrent or migratory thromboembolic events. Although frequently linked to adenocarcinomas, its association with endometrioid ovarian carcinoma in young patients is infrequently documented in the literature, posing significant diagnostic and therapeutic challenges. We report the case of a 29-year-old woman with no prior medical history who presented with acute deep venous thrombosis, bilateral pulmonary embolism, and subsequent arterial thrombotic events, including multiple ischemic strokes. Further work-up demonstrated a left adnexal lesion classified as O-RADS (Ovarian-Adnexal Reporting and Data System) 5 in the absence of thrombophilia or antiphospholipid antibodies. Transthoracic echocardiography identified a mitral valve sessile mass consistent with non-bacterial thrombotic endocarditis (NBTE). Exploratory laparotomy confirmed an endometrioid ovarian carcinoma (FIGO (International Federation of Gynecology and Obstetrics) stage IC2) arising from endometriosis. This case highlights the complexity of hypercoagulability associated with occult malignancy and underscores the importance of considering an underlying neoplasm in young patients with unexplained venous or arterial thrombosis. Trousseau’s syndrome should be included in the differential diagnosis of recurrent or idiopathic thrombotic events in young individuals. In cases of unexplained or recurrent thrombosis, clinicians should consider a tailored oncologic work-up to investigate potential underlying malignancies and improve clinical outcomes.

## Introduction

The malignancy-associated hypercoagulable state, known as Trousseau’s syndrome, was first described by Armand Trousseau in 1865 [1]. This disorder represents a prothrombotic state linked to malignant neoplasms and constitutes a classic paraneoplastic syndrome; thrombotic manifestations may involve both the arterial and venous systems and can affect critical organs such as the lungs and the central nervous system [2].

In some cases, non-bacterial thrombotic endocarditis (NBTE) coexists, occurring more frequently in lung, pancreatic, and ovarian cancers. A retrospective cohort study conducted by Patrzalek et al. involving 115 patients with cancer-associated NBTE reported a prevalence of gynecological cancers of 14.8%. Its presence is associated with poor clinical outcomes; in the same cohort, the mortality rate reached 77.9% at two years [3].

Patients tend to be asymptomatic; however, when present, symptoms are related to systemic emboli affecting the central nervous system, coronary arteries, kidneys, spleen, skin, and extremities. In some cases, even heart failure or valve dysfunction may occur. This syndrome may precede the oncologic diagnosis, particularly in adenocarcinomas, whereas in non-adenocarcinoma cancers, they typically appear during advanced stages of the disease [4].

Trousseau’s syndrome is predominantly associated with adenocarcinomas and clear cell tumors; however, its relationship with endometrioid-type carcinomas is exceptional: in a series of 827 ovarian cancers described by Takano et al., only 3.2% (27 cases) presented a thrombosis associated with Trousseau's syndrome, and only three cases (0.36% of total) corresponded to endometrioid tumors [5]. We report the case of a woman younger than 30 years, with no gynecologic-obstetric or oncologic history, who developed multiple arterial and venous thromboses, ultimately leading to the diagnosis of endometrioid ovarian carcinoma.

## Case presentation

A 29-year-old nulliparous woman with no significant chronic-degenerative or family history presented with a one-month history of sudden-onset burning pain in the left lower limb, rated 8/10 in intensity, accompanied by progressive edema that limited ambulation. Forty-eight hours later, she developed cough, hemoptysis, dyspnea, and tachycardia, prompting medical evaluation where she was found normotensive (108/76 mmHg), with sinus tachycardia (121 bpm) and a decrease in oxygen saturation to 83% on room air.

**Figure 1:**
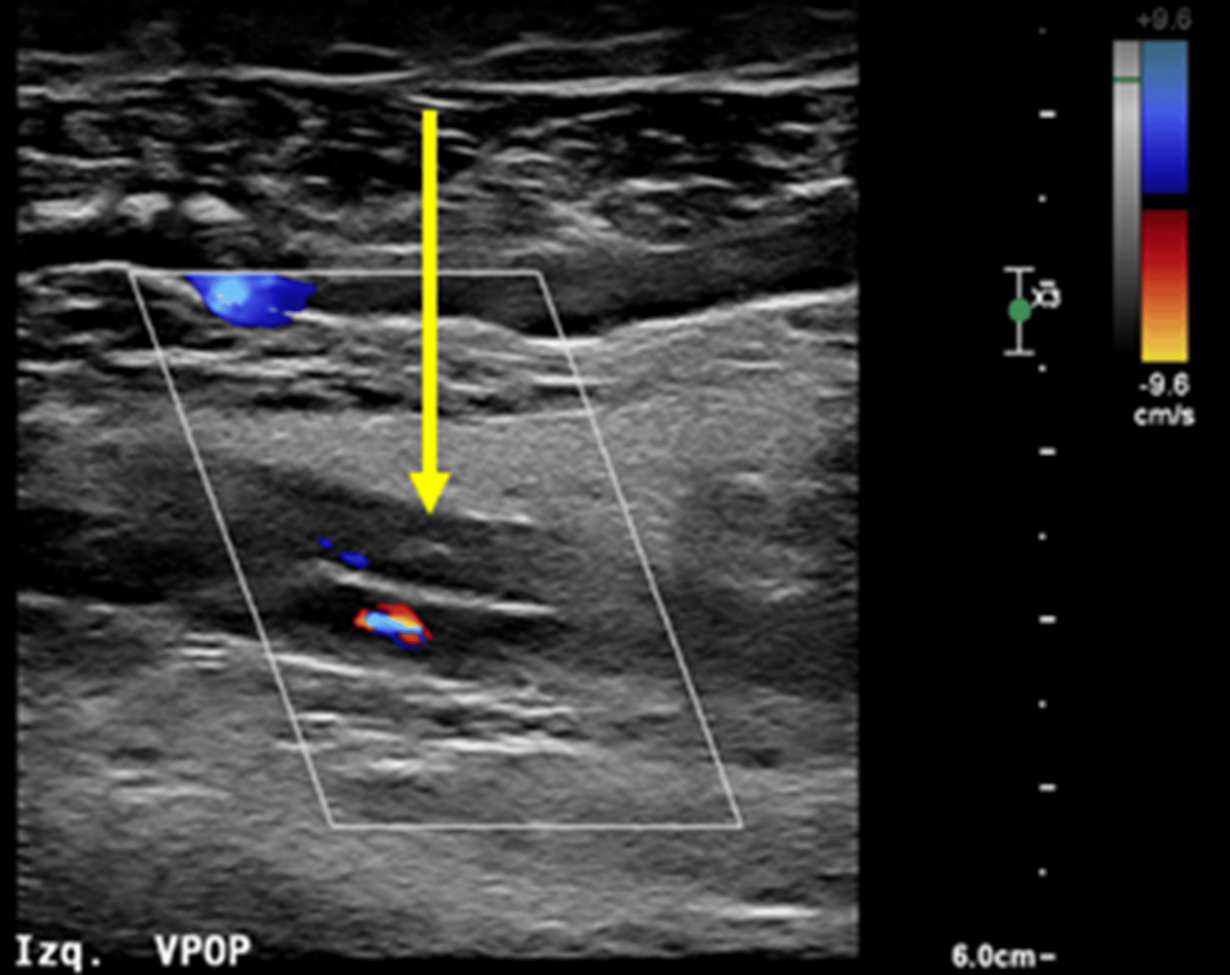
Lower limb ultrasound The arrow indicates a hyperechogenic thrombus within the left popliteal vein. Duplex Doppler examination confirms the absence of flow.

**Figure 2:**
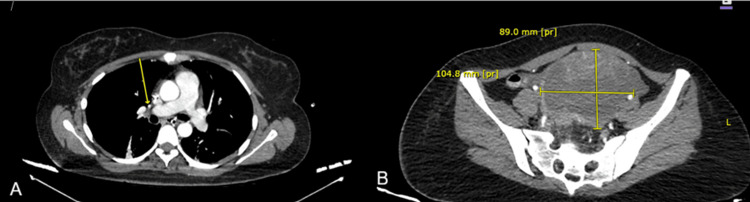
Right pulmonary thromboembolism and pelvic mass (A) Axial chest computed tomography (CT) showing a filling defect in the right pulmonary artery (arrow), consistent with pulmonary thromboembolism. (B) Axial pelvic CT revealing a large pelvic mass of indeterminate etiology.

Given the suspicion of thrombosis, D-dimer was obtained, reporting 9,000 ng/mL, and Doppler ultrasound of the left lower limb demonstrated deep vein thrombosis involving the left femoropopliteal segment and tibial veins, superficial vein thrombosis of the right great saphenous vein from the proximal third of the thigh to the ankle, and thrombosis of the right medial gastrocnemius veins (Figure 1). Contrast-enhanced thoracoabdominopelvic computed tomography (CT) revealed bilateral pulmonary artery thromboembolism, as well as a pelvic mass of indeterminate etiology (Figure 2). The patient was admitted to an external facility for placement of an inferior vena cava filter and bilateral pulmonary artery thrombectomy via pharmacomechanical thrombolysis one week after symptom onset. She was discharged five days after the procedure on rivaroxaban 20 mg every 24 hours, as the diagnosis of malignancy had not yet been confirmed, and the patient was initially treated for venous thromboembolism of undetermined etiology.

**Figure 3:**
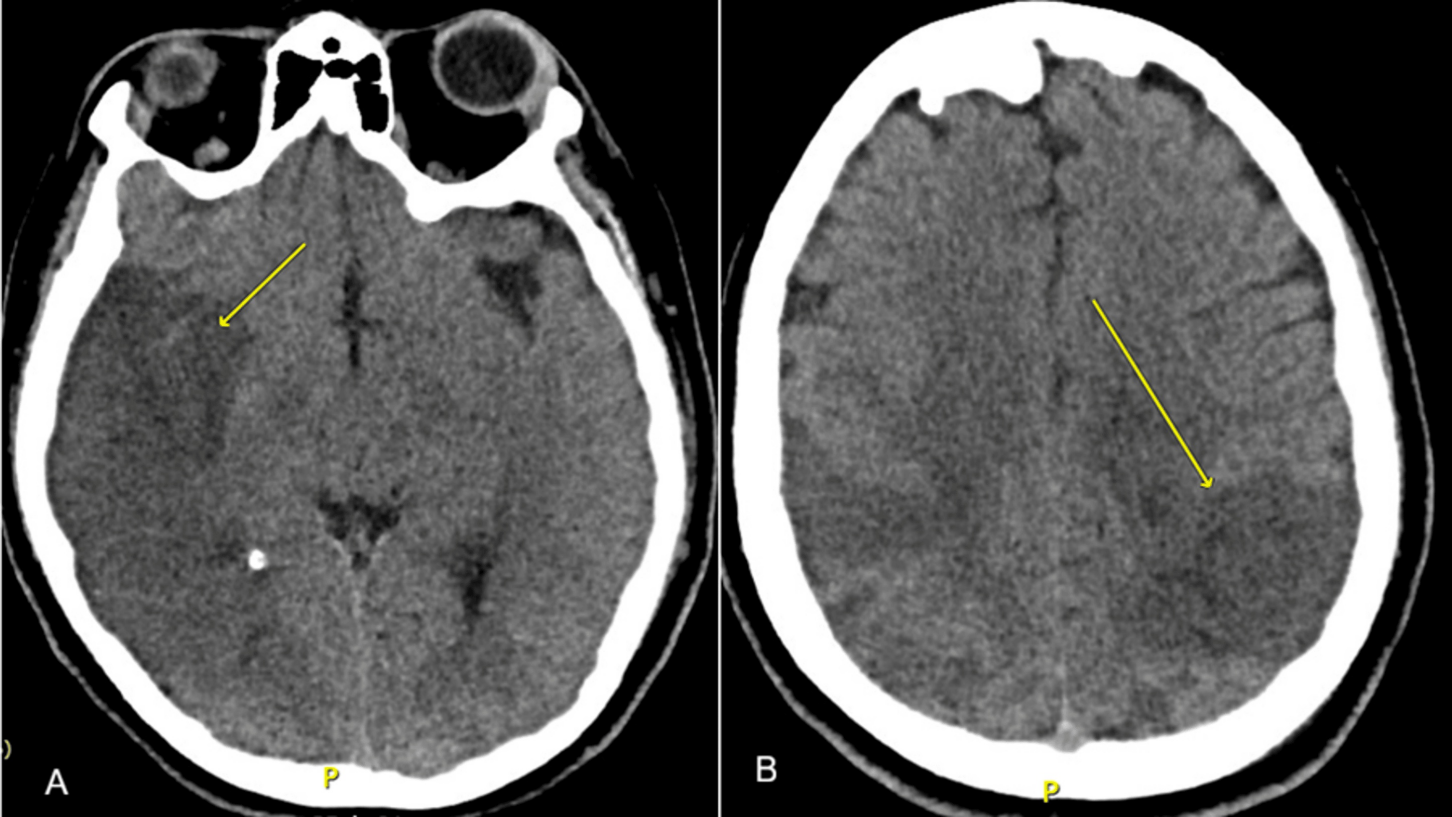
Brain computed tomography (CT) (A) Non-contrast axial cranial CT showing ischemic changes in the right parietotemporal region (arrow). (B) Ischemic area in the left parietal lobe (arrow), associated with cerebral edema.

Ten days after discharge, she experienced a generalized tonic-clonic seizure lasting five minutes, without a postictal period, followed by left hemiparesis and rightward deviation of the oral commissure, for which she was transferred to our institution. Brain CT revealed multiple ischemic areas, severe cerebral edema with midline shift, and subarachnoid hemorrhage (Figure 3), the latter constituting a contraindication for thrombolysis. Due to neurological deterioration, endotracheal intubation was performed for airway protection, and a diagnostic work-up for thrombosis in a young patient was initiated.

Given the previously noted indeterminate pelvic lesion, a repeat pelvic ultrasound was performed, reporting a left adnexal mass classified as O-RADS (Ovarian-Adnexal Reporting and Data System) 5, indicating a >50% probability of malignancy. Using the IOTA (International Ovarian Tumor Analysis) model, the risk of malignancy was estimated at >50%, prompting contrast-enhanced cervicothoracoabdominopelvic CT, which demonstrated a complex left adnexal mass containing a solid component with heterogeneous contrast enhancement, measuring 103 × 91 × 99 mm, with an estimated volume of 424 cc.

Laboratory evaluation showed negative anti-β2-glycoprotein antibodies, lupus anticoagulant, and anticardiolipin IgG and IgM. Tumor markers and immunological profile were obtained as shown in Table 1. Thrombophilia studies, including prothrombin gene mutation (G20210A) and factor V Leiden, were negative, and coagulation times remained within normal ranges. As she did not meet the clinical classification criteria for rheumatologic diseases associated with thrombosis such as systemic lupus erythematosus or antiphospholipid antibody syndrome, the isolated serologic findings were interpreted as an epiphenomenon, with the remaining findings suggesting a neoplastic etiology.

**Table 1:** Summary of tumor markers and immunological profile

Parameter	Result	Reference range
Tumor markers		
Cancer antigen 125 (CA-125)	748 U/mL	1-35 U/mL
Cancer antigen 19-9 (CA 19-9)	60.5 U/mL	<34 U/mL
Cancer antigen 15-3 (CA 15-3)	104 U/mL	<26.2 U/mL
Alpha-fetoprotein (AFP)	2.43 ng/mL	<8.1 ng/mL
Beta-human chorionic gonadotropin (β-hCG)	<0.20 mIU/mL	<2 mIU/mL
Immunological profile		
Antinuclear antibodies (ANA) by immunofluorescence	1:160	<1:40
Anti-double-stranded DNA (anti-dsDNA)	47.2 IU/mL	<30 IU/mL
Complement C3	148 mg/dL	90-180 mg/dL
Complement C4	27.20 mg/dL	10-40 mg/dL

**Figure 4:**
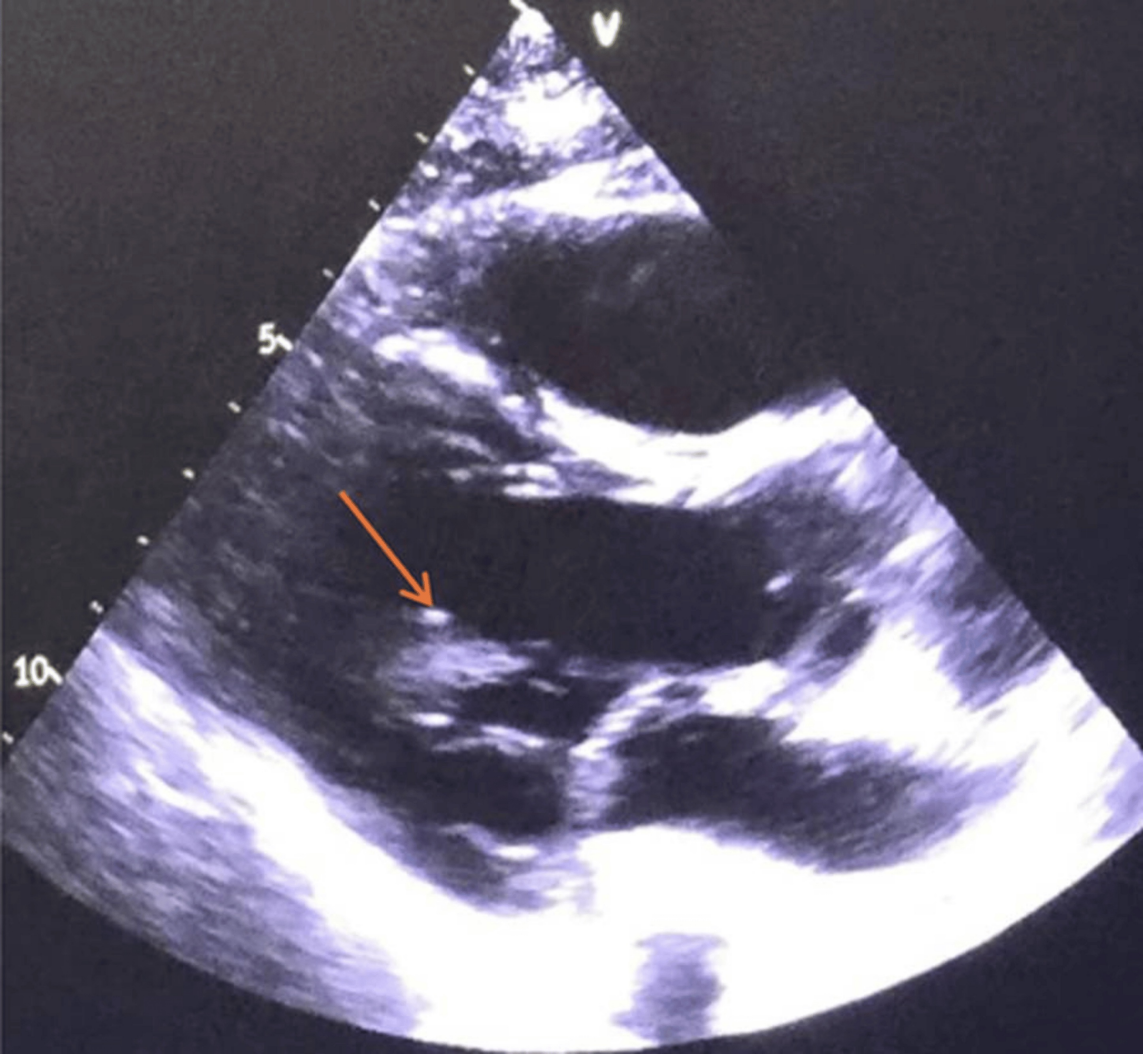
Parasternal long-axis (PLAX) view on transthoracic echocardiogram The echocardiogram image demonstrates voluminous, irregular, and echogenic masses (vegetations) attached to the atrial surface of the mitral valve leaflets and the aortic valve apparatus.

As part of the cerebrovascular event protocol, transthoracic echocardiography was performed, revealing a sessile mass attached to the atrial surface of the posterior mitral leaflet (4 × 5 mm) (Figure 4). Central and peripheral blood cultures were negative; furthermore, the patient remained afebrile throughout her hospitalization and did not develop leukocytosis, with a white blood cell count consistently within normal limits. These findings, along with a negative procalcitonin test, supported the diagnosis of NBTE over an infectious etiology. Transesophageal echocardiography was not performed since additional imaging was not considered necessary, as it was unlikely to modify the diagnostic impression or immediate management.

**Figure 5:**
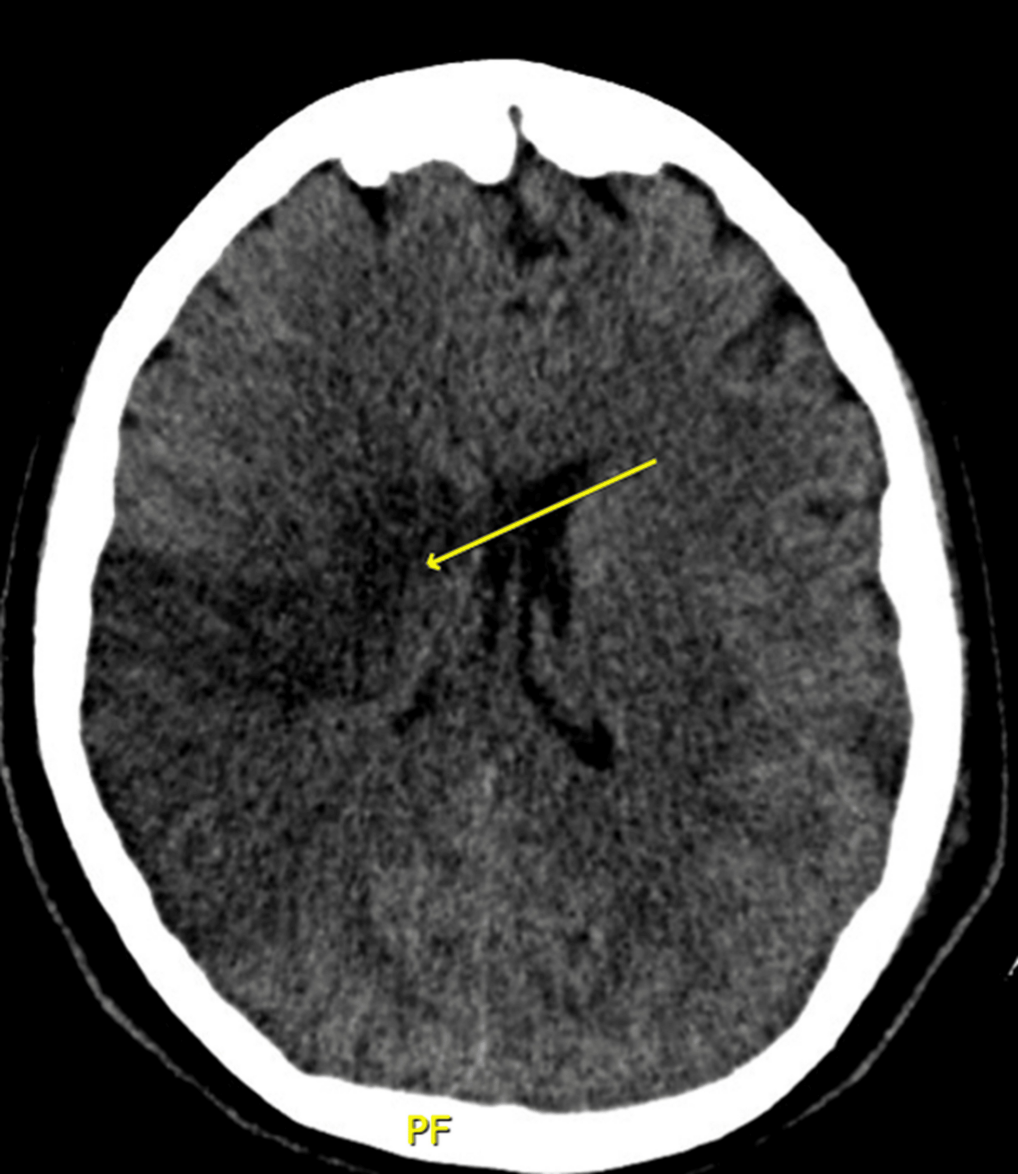
Control brain computed tomography (CT) Brain CT demonstrating a large hypodense area involving the right frontoparietotemporal region (arrow), with associated cytotoxic edema.

Follow-up brain CT performed 48 hours after the initial study demonstrated multiple subacute ischemic events: in the right middle cerebral artery (M1 segment) and the left middle cerebral artery (M4 segment), with cytotoxic edema and an 18 mm midline shift (Figure 5). In the absence of hemorrhage, therapeutic-dose low-molecular-weight heparin (LMWH) was initiated.

**Figure 6:**
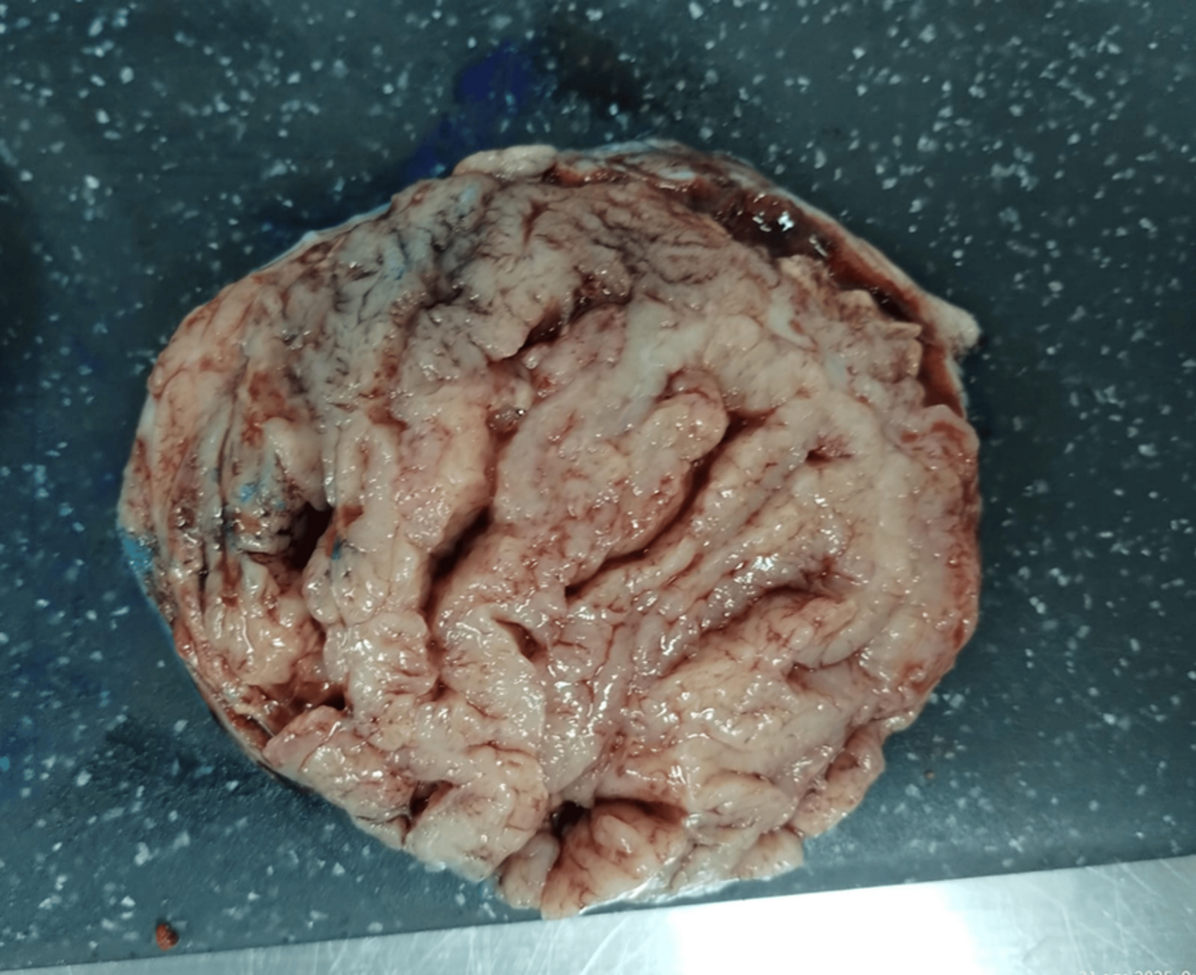
Left ovarian tumor measuring 10.5 x 8.5 x 7 cm

Given the pelvic mass of uncertain behavior with high suspicion of malignancy, dysgerminoma was ruled out based on tumor markers, and the absence of metastases led to the decision that she was not a candidate for cytotoxic therapy; instead, surgical management for mass resection was chosen. She underwent exploratory laparotomy, which revealed a left ovarian tumor measuring 100 × 120 mm, with capsular rupture and chocolate-colored content adherent to the posterior uterine wall (Figure 6). Intraoperative frozen section analysis was positive for malignancy, leading to total abdominal hysterectomy, bilateral salpingo-oophorectomy, and omentectomy. These findings correspond to FIGO (International Federation of Gynecology and Obstetrics) stage IC2 ovarian cancer.

The final histopathological report concluded an endometrioid carcinoma arising from a focus of endometriosis. No lymphovascular invasion or involvement of the ovarian surface was identified. The contralateral ovary was negative for neoplasia, bilateral pelvic lymph nodes showed reactive hyperplasia without neoplastic infiltration, and the omentum demonstrated vascular congestion without tumor involvement.

Postoperatively, the patient remained hemodynamically stable and was discharged with a tracheostomy, direct oral anticoagulation, and follow-up with internal medicine and rehabilitation. At subsequent evaluations, no new thrombotic events were documented.

## Discussion

This case highlights the complex association between malignancy and a prothrombotic state, emphasizing that an occult malignancy should be considered in any patient with unexplained or recurrent thrombotic events. Cancer-related hypercoagulability, also known as Trousseau’s syndrome, is characterized by the production of procoagulant factors by tumor cells, such as coagulation factors (VII, VIII), tissue factor, and cancer procoagulant [6,7]. This syndrome, first described by Armand Trousseau (1801-1867) in 1865, is also associated with multiple medical descriptions and manifestations [8]. He died in 1868, describing symptoms of “phlegmasia alba dolens” secondary to stomach cancer. Subsequently, Sack in 1977 added manifestations to this syndrome, such as aseptic endocarditis, microangiopathy, arterial embolisms, and disseminated intravascular coagulation. Although this syndrome is relatively uncommon, it has been reported in several malignancies such as gastric, pancreatic, lung, and ovarian cancer [9,10].

The risk factors associated for cancer thrombosis can be divided into three categories: patient factors such as age, immobility, previous history of thrombosis, elevated platelet or leukocyte count, and obesity; treatment-related factors such as hormonal therapy, growth factor, and antiangiogenic factors; and cancer-specific factors, such as primary site, higher stages, direct vascular compression or invasion, mucin-producing adenocarcinoma, and tissue factor expression. Therefore, in cases of ovarian cancer, the expression of cancer antigen 125 (CA-125) is a marker of thrombosis risk as it is associated with increased mucin [11]. Regarding other biomarkers in patients with Trousseau’s syndrome, D-dimer (as in our patient) and CRP levels were higher than in patients with other causes of thrombosis such as heart disease or atheromatosis [12].

In our patient, the initial manifestation was an ischemic stroke, an arterial presentation of this syndrome, that, while possible, is less frequently described in the literature [13,14]. When cerebrovascular disease occurs, bilateral infarction is more typical of Trousseau’s syndrome than cardiogenic or artery-to-artery embolisms [15]. In the presence of widespread thrombosis, patent foramen ovale or intracavitary thrombi must be ruled out by echocardiography.

The frequency of ovarian cancer in Trousseau’s syndrome has historically been reported at 3.8%, with the clear cell subtype being the one with the highest risk [16], and the association between ovarian cancer and stroke is 1.1% to 1.7% [5]. Another thrombotic complication in this case was NBTE, occurring in 0.9%-1.6% of autopsies on cancer patients, being more frequent in solid tumors (2.7%) than in non-solid tumors (0.47%). It consists of sterile vegetations in cardiac valves, composed primarily of fibrin and platelets, which can embolize to systemic circulation causing ischemic stroke, as seen in this patient [17]. The most affected valves are the aortic valve, the mitral valve, and a combination of both [18,19]. Even though it has been described in antiphospholipid syndrome and other rheumatologic conditions such as systemic lupus erythematosus, it has also been described in many malignancies [19], particularly in association with ovarian cancer [4].

Heparin remains effective in the acute treatment and prophylaxis of thromboembolism in Trousseau's syndrome. LMWH is associated with fewer major bleeding complications than unfractionated heparin, while warfarin has been reported to be less effective in this population, with increased mortality [20]. In recent years, several randomized clinical trials have evaluated direct oral anticoagulants (DOACs) in cancer-associated thrombosis (CAT). The Hokusai-VTE Cancer and SELECT-D trials demonstrated that edoxaban and rivaroxaban were non-inferior to LMWH in preventing recurrent venous thromboembolism, although with a higher incidence of bleeding in selected patients [21,22]. More recently, the CARAVAGGIO trial showed that apixaban was non-inferior to dalteparin without a significant increase in major bleeding [23]. Based on these findings, current guidelines from the American Society of Hematology (ASH) and the American Society of Clinical Oncology (ASCO) support the use of DOACs as appropriate options for the treatment of cancer-associated venous thromboembolism in selected patients [24,25]. However, evidence specifically addressing DOAC use in malignancy-associated NBTE remains limited, and anticoagulation strategies should be individualized according to clinical context and bleeding risk.

Our patient’s clinical course was particularly severe due to neurological compromise that required mechanical ventilation, followed by complex surgical management involving hysterectomy and bilateral oophorectomy. Despite the diagnostic and therapeutic challenges, the patient achieved a favorable clinical recovery. Prognosis in patients with Trousseau’s syndrome associated with stroke is heterogeneous and largely influenced by tumor type, stage, and overall oncologic burden. Some retrospective series involving patients with advanced malignancy have reported a median survival of approximately 4-5 months, with early mortality rates approaching 25% within 30 days of diagnosis; however, these outcomes should not be generalized to all patients [5,13].

In summary, this case reinforces the association between ovarian malignancy and a hypercoagulable state. While predominantly observed in older populations, this case underscores that in any patient, regardless of age, with unexplained or recurrent events of thrombotic phenomena, an occult malignancy should be strongly considered, especially in the presence of non-bacterial valvular vegetations.

## Conclusions

This case illustrates the diagnostic and therapeutic challenges posed by Trousseau’s syndrome in the context of endometrioid ovarian carcinoma in a young patient. The clinical course was characterized by successive venous and arterial thromboembolic events, as well as severe neurological compromise, all occurring in the absence of identifiable rheumatologic disease or inherited thrombophilia. Early recognition of these thrombotic manifestations-particularly in young patients without apparent risk factors-is essential for timely identification of the underlying neoplasm and for improving outcomes through targeted interventions. This case reinforces the need for a multidisciplinary approach that includes comprehensive imaging evaluation, tumor biomarker assessment, and histopathological confirmation to detect and treat the underlying neoplasm at earlier stages. It also highlights the importance of maintaining a high index of suspicion when faced with idiopathic or recurrent thrombotic events in young individuals, suggesting that further studies are warranted to evaluate the potential benefit of standardized oncologic investigation protocols in such scenarios.
